# The Response of Substrate Microbial Communities to the Addition of Mineral Nutrients During the Growth Period of Straw Mushroom *Volvariella volvacea*

**DOI:** 10.3390/microorganisms14010056

**Published:** 2025-12-26

**Authors:** Le Wang, Yan Zhao

**Affiliations:** Institute of Edible Fungi, Shanghai Academy of Agricultural Sciences, Shanghai 201403, China; wangle202108@126.com

**Keywords:** *Volvariella volvacea*, nutrient, microbial diversity, metabolism

## Abstract

*Volvariella volvacea* were grown on an abandoned cotton-based substrate, which was divided into two conditions: a group with added nutrients (N3P3) and a control group (CK). Using metagenomic sequencing technology, the study investigated the effect of nutrient addition during the growth process of *V. volvacea* on the microbial community and metabolic pathways of the substrate. The study found that the main bacteria in the N3P3 group were *Proteus* and *Microsporidium*, while in the CK group, *Bacillus marinosus and Microsporidium globosa* were more common. At all stages of *V. volvacea* growth, *Proteobacteria* and *Firmicutes* dominated. Metabolic function analysis showed that the N3P3 group significantly increased amino acid metabolism, nitrogen metabolism, genetic information processing, and cellular processes, while reducing the contents of pathogenic and saprophytic symbiotic fungi. Nitrogen metabolism, phosphorus metabolism, and carbon metabolism were closely related to the growth of *V. volvacea*, and nutrient addition significantly improved microbial community diversity and metabolic levels, which can be used as a substrate optimization formula. This is of great significance for the development of sustainable agriculture.

## 1. Introduction

The annual output of edible fungi in China is more than 45 million tons, accounting for 94.01% of the world’s total output, and the characteristic mushroom *Volvariella volvacea* is becoming increasingly popular. It contains an appropriate amount of high-quality protein, which is a good source of dietary fiber, vitamin C, B vitamins, and minerals [1,2,3]. *V. volvacea* grows on a variety of substrates, and cotton waste is the most commonly used [4]. Mineral supplementation can improve the yield of *V. volvacea* [5]. Adding nitrogen and phosphorus nutrients to the substrate can significantly improve the yield and biological efficiency of edible fungi [6].

However, excessive nitrogen and phosphorus levels will inhibit the yield of *V. volvacea* [7]. Environmental factors interact with microorganisms, jointly influencing the growth of *V. volvacea*. Adding nutrients can indirectly lead to changes in growth, metabolic processes, and community composition of microorganisms through altering the physicochemical properties and nutrient content of the substrate. On the contrary, nutrients, as metabolic substrates of enzymes, can also influence the activity of enzymes. In addition, microorganisms will participate in the decomposition and transformation of organic matter, and alterations in their composition can result in changes in their metabolism, mortality rates, and physicochemical properties [8]. Microorganisms in the substrate exert a vital role in energy flow and nutrient cycling and are involved in organic matter decomposition, exogenous biodegradation, and the prevention and control of edible fungus pathogens [9,10].

Therefore, this study aims to reveal the molecular mechanism by which the diversity of substrate microorganisms promotes the growth of *V. volvacea* by nutrient regulation in the substrate. This study adopts metagenomic methods in order to better understand the dynamic changes in substrate microorganisms during the growth of *V. volvacea*. Therefore, this study aims to explore the effect of nutrient conditions on the substrate microbial community during the growth process of *V. volvacea*. The interaction mechanism between *V. volvacea* and microbial communities were explored at the micro level.

## 2. Materials and Methods

### 2.1. Cultivation Method and Sampling

The work was conducted at the ZhuangHang Experimental Base of the Shanghai Academy of Agricultural Sciences. *V. volvacea* was provided by the Shanghai Fanshun Edible Fungi Professional Cooperative. Waste cotton was chosen as the culture material for *V. volvacea* cultivation. In this work, substrate samples were selected at four growth stages of *V. volvacea* to investigate dynamic changes in the composition of the substrate microbial community during the growth of *V.volvacea*. The substrate contains 5% lime, 10% wheat bran, and 85% waste cotton. The nitrogen and phosphorus contents in the CK and N3P3 treatment groups were CK (0, 0) and N3P3 (75 g urea and 30 g diammonium phosphate), respectively. AN, AD, AS, and AC represent the button stage, oval stage, elongation stage, and maturation stage, respectively; N3P3N, N3P3D, N3P3S, and N3P3C represent the button stage, oval stage, elongation stage, and maturation stage after adding N3P3, respectively.

### 2.2. Metagenomic DNA Extraction and Shotgun Sequencing

Upon arrival at the laboratory, each sample was immediately preserved at −80 °C. DNA extraction was completed with MagBeads FastDNA Kit for Soil (116564384) (MP Biomedicals, Irvine, CA, USA) according to the manufacturer’s instructions and stored at −20 °C prior to further assessment. The quantity and quality of extracted DNAs were measured using a Qubit™ 4 Fluorometer, with WiFi: Q33238 (Qubit™ Assay Tubes: Q32856; Qubit ™ 1X dsDNA HS Assay Kit: Q33231) (Invitrogen, Carlsbad, CA, USA) and agarose gel electrophoresis, respectively.

### 2.3. Metagenomics Analysis

Extracted microbial DNA was processed to construct metagenome shotgun sequencing libraries with insert sizes of 400 bp by using the Illumina TruSeq Nano DNA LT Library Preparation Kit. Each library was sequenced on a Illumina NovaSeq platform (Illumina, San Diego, CA, USA) with a PE150 strategy at Personal Biotechnology Co., Ltd. (Shanghai, China).

### 2.4. Data Processing and Analysis

Raw sequencing reads were processed to obtain quality-filtered reads for further analysis. First, adapters were removed and low-quality reads were trimmed using fastp v0.23.2 [10,11]. Then, MEGAHIT software v1.1.2 was used to concatenate and assemble the valid reads for each sample, with contigs of no less than 300 bp length retained by default. Open reading frames (ORFs) were identified using Prodigal software v2.6.3 to predict coding regions and obtain corresponding gene and protein sequence files. At the same time, redundant sequences were removed using the clustering module of mmseq2 software (MMseas v13.45111) to obtain non-redundant gene and protein sets. Upon obtaining quality-filtered reads, taxonomic classification of each sample was executed using Kraken2 with the “--confidence 0.5” option against a customized database containing nucleotide sequences of prokaryotic and eukaryotic microorganism from NCBI-nt, and viruses from RVDB.

For the obtained non-redundant protein set, species annotation was performed using the mmseq2 software (MMseas v13.45111). The database contains protein sequences of prokaryotic and eukaryotic microorganisms from NCBI-nr and viruses from RVDB. Use the Strobealign plugin to map the high-quality reads of each sample onto the overlapping group, and use the featureCounts to count reads to determine gene abundance. Using mmseq2 in “search” mode with the parameter “-s 5.7”, functional number annotations were performed on databases such as KEGG, eggNOG, GO, CAZy, and CARD to obtain species abundance tables at various classification and functional levels [12,13,14,15,16,17].

All data are expressed as means of three independent replicates. Statistical analyses were carried out with R (version 4.3.3). Differences were considered statistically significant at *p* < 0.05. ASV-level alpha diversity indices, including the Simpson index, were calculated using the ASV table in QIIME2. LEfSe (linear discriminant analysis effect size) was performed to detect differentially abundant taxa across groups using the default parameters [18,19].

## 3. Results and Discussion

### 3.1. Species-Level Qualitative Assay

**Figure 1:**
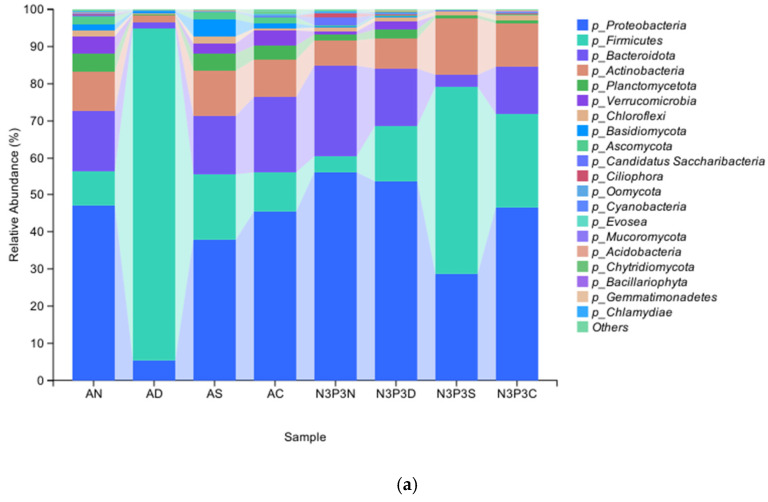

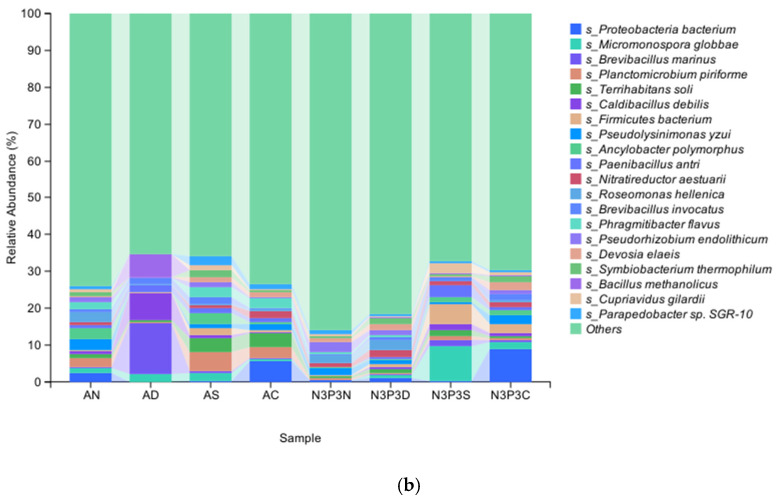
(**a**) Analysis of the composition of the top 20 microbial species at the phylum level. (**b**) Analysis of the composition of the top 20 microbial species at the species level. Unfertilized samples (AN: button stage, AD: egg-shaped stage, AS: elongation stage, and AC: maturity stage). Fertilization samples (N3P3N: button stage, N3P3D: egg-shaped stage, N3P3S: elongation stage, and N3P3C: maturity stage).

The composition of substrate microbial communities was analyzed at four growth stages of *V. volvacea* before and after nutrient addition. After filtering and quality control, the total valid metagenomic sequencing data volume was 989,712,102 reads, with an average q30 of 94% per sample (Figure 1). From the different groups (AN, AD, AS, AC, N3P3N, N3P3D, N3P3S, and N3P3C) 22 phyla and 526 genera were identified in 15,426 ASVs. More than 35% of ASVs were classified at the species level of the microbiota. During each growth stage (the stage without nutrient addition and the stage with nutrient addition), the dominant microbial communities were composed of *Proteobacteria*, *Firmicutes*, *Bacteroidota*, *Actinobacteria,* and *plantomycetota*. Under both conditions across the four stages, *Proteobacteria*, Firmicutes, and Bacteroidota exhibited similar trends of changes in microbial diversity, showing an increase first followed by a decrease. During the egg-shaped period under CK conditions, Firmicutes was the most abundant, accounting for 89.73%. However, after adding nutrients, it was gradually replaced by *Proteobacteria*. In the mature period under both conditions, *Proteobacteria* was the most abundant, although its relative abundance decreased gradually over time compared with the egg-shaped period. Under nutrient addition, the abundance of *Proteobacteria* (56.15%) was higher than that in the CK group (47.23%).

Among all groups, *Proteobacteria* and Firmicutes were the microbial communities with the highest relative abundance, followed by Bacteroidetes and Actinomycetes. Under nutrient addition treatment, *Proteobacteria* were relatively abundant in the early growth stages, but during the extension period, Firmicutes became the dominant species. Under the treatment without nutrient addition, the relative abundance of basidiomycetes gradually increased from the early growth stage, reaching the highest relative abundance at the extension stage (Figure 1a). In both the treatments without nutrient addition and with nutrient addition, *Proteobacteria* and Firmicutes were dominant. The relative abundance of *Proteobacteria* showed a trend of first decreasing and then increasing from the early growth stage to the end of the growth period of *V. volvacea*, whether in the treatment without nutrient addition or with nutrient addition. In contrast, *Firmicutes* showed a trend of first elevating and subsequently lowering. At the species level (Figure 1b), the relative abundance of microorganisms treated with nutrient addition in the microbial community structure among each group increased by approximately 20% compared to those treated without nutrient addition. In each treatment, the abundance of *Proteobacteria* gradually increased under nutrient addition treatment. Among them, the relative abundance of small monospora in the nutrient treatment increased by 5% compared with that in the non-nutrient addition treatment. *Proteus* and *Pseudomonas aeruginosa* became the dominant bacterial communities under nutrient addition. 

There have been numerous studies on the microorganisms and metabolic processes during the growth stages of edible fungi [20,21,22,23,24,25]. Research shows that during the elongation stage of *V. volvacea*, the abundance of many bacteria (such as *Bacteroides*, *Acidobacteria*, and *Nitrosomonas*) increases significantly [26]. In the mature stage, these bacteria are key participants in the degradation of the *V. volvacea* substrate [27], facilitating the decomposition, transformation, and metabolism of substrate materials [28]. Among them, *Proteobacteria* and *Actinobacteria* dominate the *V. volvacea* growth process [29,30]. The main functions of the *Proteobacteria* phylum and *Micrococcus* are to carry out oxidation reactions to remove pollutants, which is beneficial for the growth and development of *V. volvacea* [31] and enhances fruiting body yield [32]. This study found that with and without nutrient addition, the abundance of *Proteobacteria* and *Actinobacteria* in the *V. volvacea* substrate was the highest, at 47.23% and 45.56%, respectively, and 56.15% and 53.74%, respectively. The research results indicate that when nutrients are added, the abundance of *Proteobacteria* and *Actinobacteria* is highest. When no nutrients are added, the abundance of *Proteobacteria* and *Micrococcus* gradually increases, indicating that these microorganisms are dominant populations. This research results are consistent with the above research conclusions.

### 3.2. Classification of Microbial Composition According to Analysis

**Figure 2:**
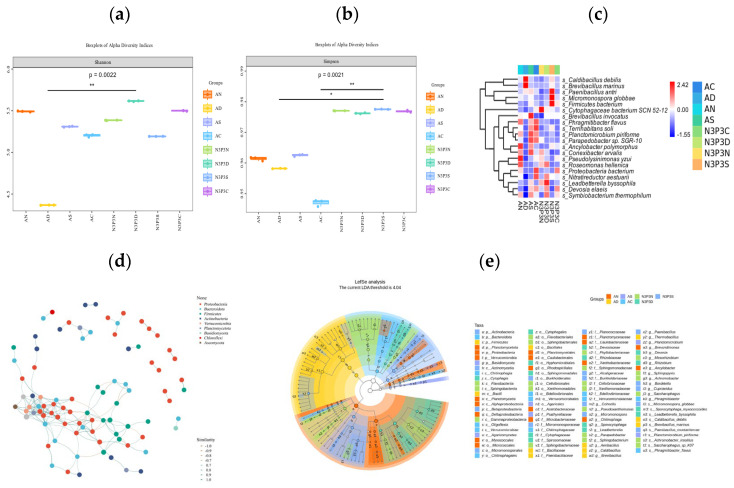
Boxplots of alpha diversity indices: Shannon index (**a**); Simpson index (**b**); Simpson’s index (**c**); Lefse analysis of different species (**d**); associative network diagram (**e**). In the figure, a post-hoc test was conducted between the two groups. The significant marks of the Dunn’s test post-hoc test are plotted, using horizontal lines and * symbols to indicate the results of the post-hoc tests for the two groups. * represents *p* < 0.05, and ** represents *p* < 0.01.

Figure 2a shows the relative abundances of the top 20 species of microorganisms in the substrate during the growth process of *V. volvacea*. The results revealed that in the no-nutrient addition group, the abundance of anylobacter *Polymorphus*, *Conexibacter arvalis*, *pseudolysimonas yzui*, *Roseomonas, hellenica* and *Phragmitibacter flavus* increased. These beneficial bacteria can interact with the host, provide nutrients to the host, and promote the production of *V. volvacea* [33]. In the nutrient addition group, *Bacillus gastroenteritis*, *Trichosporum spheroidium*, *Pachyphylum*, *Lead acidophilus,* and *Devosia elaeis* were more abundant. These microorganisms usually serve as functional extensions of the host genome and exert a vital role in the regulation of host physiology and metabolism [34].

Nitrogen and phosphorus are significantly related to the yield and biological efficiency of edible fungi [35,36]. Nitrogen sources not only promote the synthesis of enzymes, but supplementation with nitrogen can also increase the nutritional content of the substrate [37], thereby increasing the yield of edible fungi [38]. Studies on bacterial community structure of the substrate and its interaction with environmental factors revealed that *Firmicutes* are positively correlated with total organic carbon, nitrogen, and phosphorus content [39], and that the addition of nutrients increased the abundance of *Acidobacteria* and *Firmicutes* [40]. This study found significant differences in the Shannon index of the microbial community in the *V. volvacea* substrate under two nutrient conditions (*p* = 0.0022). In the egg-shaped stage of the *V. volvacea* with added nutrients, microbial diversity of the substrate was significantly different from that in the group without added nutrients (*p* < 0.01) (Figure 2b). The results of the Simpson index indicated significant differences among microbial communities across groups (*p* = 0.0021), which were consistent with previous research results [41,42,43]. At the same time, regardless of whether nutrients were added or not, with the extension of the growth period, changes in the microbial community structure showed a trend of first decreasing and then increasing. This indicates that the addition of nutrients significantly increased the microbial diversity in the *V. volvacea* substrate (Figure 2c).

In this study, the dominant microbial species at the ACKDX and N3P3SHC stages were *Micromonospora globbae*, *Caldibacillus debilis*, and *Brevibacillus marinus*. At the N3P3CHS, N3P3DX, and N3P3NQ stages, the dominant microbial species were *Pseudolysinimonas yzui*, *Roseomonas hellenica*, and *Proteobacteria.* However, at the ACKNQ, ACKSHC, and ACKCHS stages, the dominant microbial species were *Ancylobacter polymorphus*, *Terrihabitans soli*, *Planktomicrobium piriforme*, and *Phragmitibacter flavus.* These results indicate that *Proteobacteria* play a role in promoting the decomposition of substrate lignin and the development of *V. volvacea* fruiting bodies [44]. *Ancylobacter polymorphus* promotes the metabolism of intermediate products, *Caldibacillus debilis* promotes cellulose decomposition, and *Proteobacteria* are involved in nitrogen fixation, carbon–nitrogen cycling, and maintenance of substrate micro-ecosystem balance.

LEfSe analysis of different microbial species indicated that there were seven distinct microbial communities at the phylum level (Figure 2d), including *Actinobacteria*, *Bacteroidetes*, and *Firmicutes*, among which *Firmicutes* and *Actinobacteria* had the highest abundance in AD and N3P3S. At the species level, there were a total of 10 different microorganisms, mainly including *Micromonospora globbae*, *Caldicibacillus deteris*, and *Brevibacillus marinus*, among which *Caldicibacillus deteris* and *Micromonospora globbae* had the highest abundance in AD and N3P3D groups. These findings demonstrated that increased microbial diversity enhances carbon utilization efficiency, improves substrate physical properties, alters microbial community composition in the substrate, increases the relative abundance of microorganisms, and provides substrate nutrition, thereby effectively increasing the yield of *V. volvacea* [45,46,47,48,49].

*Proteobacteria* can convert substrate substances into main nutritional sources for mycelium, promoting the growth of mycelium during the cultivation process [50]. To better understand the classification characteristics of the microbial community, we conducted a network analysis. The results of the network analysis showed that there were 205 edges among 87 nodes, indicating significant correlation among microbial communities. In the co-occurrence network, high-abundance nodes were classified into nine microbial phyla, with *Proteobacyeria*, *Bacteroidota*, *and Firmicutes* accounting for 70% of all nodes. *Proteobacteria* was the one with the most nodes in the association network (Figure 2e), indicating its dominant role in the substrate microbial community and its strong associations with other microorganisms.

### 3.3. Functional Analysis

**Figure 3:**
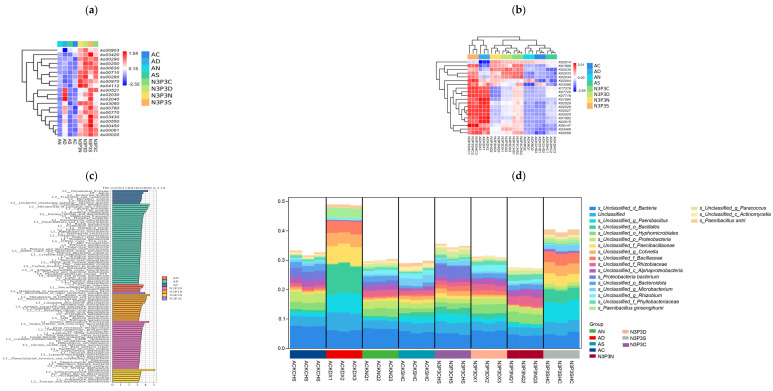
Heatmap of the abundance of metabolic pathways at the KEGG L3 level (**a**); KEGG differential functional gene heatmap (**b**); KEGG analysis (**c**); analysis of the functional contributions of species (**d**).

In this study, the KEGG pathway analysis revealed that microbial metabolic pathways were significantly enriched in carbon metabolism, environmental information processing, and genetic information processing (Figure 3). The abundance of metabolic pathways was highest during the exponential phase and the stationary phase, followed by the lag phase (Figure 3a,b). Without nutrient addition, metabolic pathway abundance was higher during the egg-shaped phase but lower during the lag phase, exponential phase, and stationary phase (Figure 3c). With nutrient addition, metabolic pathway abundance was significantly higher than without nutrient addition (Figure 3d). Metabolic pathways without nutrient addition mainly included long-chain fatty acid degradation, energy and lipid metabolism regulation, the pyruvate cycle, vitamin synthesis, degradation, and function realization. In contrast, metabolic pathways with added nutrients mainly enriched the following pathways: DNA recombination, fatty acid synthesis, pyruvate formation and esterification, energy balance and lipid metabolism regulation, nucleotide synthesis and catabolism, and carbon metabolism. In summary, the addition of nutrients promoted the expression of KEGG metabolic pathways.

LEfSe analysis results showed that there were 80 different functional metabolic pathways in L3, covering pathways related to systems, transport and decomposition metabolism, bacterial chemotaxis, terpenoid and polyketide metabolism. Based on KEGG functional analysis, L3 indicated a significant difference between the group without nutrient addition and the group with nutrient addition (R^2^ = 0.94, *p* = 0.001). After adding nutrients at different growth stages, the expression of functional genes increased (Figure 3b), which might be related to substrate nutrition during the growth process of *V. volvacea* [51]. In the group with added nutrients, there were 54 highly expressed metabolic pathways, mainly including amino acid metabolism, nitrogen metabolism, genetic information processing, and energy metabolism. There were significant differences in functional gene expresssion among the groups. As the nutritional level of *V. volvacea* increased during different growth periods, the expression of functional genes also increased (Figure 3c), which had a significant impact on the potential microecology and biochemical cycling functions of the microbial community [52]. In each period of *V. volvacea* growth, protein synthesis, cell cycle regulation, carbon and energy metabolism, amino acid metabolism, oxidative phosphorylation, and lipid metabolism were the main metabolic pathways. These metabolic functions are of great significance for understanding the growth and development of *V. volvacea* during the cultivation process [53].

Analysis of species functional contribution indicated that the main bacterial groups associated with these metabolic pathways are *Proteus*, *Bacillus ginseng*, and *Pseudomonas*, with contribution rates ranging from 1% to 5%. Among them, *Proteobacteria* showed the highest contribution rate at 5%. The N3P3 group includes 54 highly expressed metabolic pathways (Figure 3d). Nitrogen, phosphorus, and carbon metabolism are closely related to the growth of *V. volvacea* [54], which can maintain an adequate energy supply and help reduce the catabolism of glutamic acid and proline [55]. These microorganisms participate in the regulation of glucose metabolism and play an important role in lowering blood sugar, providing a theoretical basis for the utilization of potential functional food components to prevent and treat diabetes.

## 4. Conclusions

In this study, during each growth stage of the *V. volvacea* (with and without nutrient addition), the dominant microbial communities consist of *Firmicutes*, *Actinobacteria*, *Bacteroidetes*, *Proteobacteria*, and *Spirochaetes*. Regardless of whether nutrients are added or not, *Bacillus* and *Actinobacteria* were the most abundant, making them the main microbial communities. They occupy dominant positions in the correlation network and have strong correlations with other microorganisms. Addition of nutrients significantly increased microbial diversity in the *V. volvacea* substrate. *Bacillus gastroenteritis*, *Trichosporum spheroidium*, *Pachyphylum*, *Lead acidophilus*, and *Devosia elaeis* were more abundant under nutrient addition. These microorganisms usually serve as functional extensions of the host genome and play a vital role in regulating the host’s physiology and metabolism. *Ancylobacter polymorphus* promotes the metabolism of intermediate products, *Caldibacillus debilis* promotes cellulose decomposition, and *Firmicutes* participate in nitrogen fixation and carbon–nitrogen cycling, thereby promoting better utilization of carbon–nitrogen sources by *V. volvacea* and increasing yield. KEGG metabolic pathways were significantly enriched in carbon metabolism, environmental information processing, and genetic information processing. The addition of nutrients promoted the expression of KEGG metabolic pathways, and at each growth stage of the *V. volvacea*, protein synthesis, cell cycle regulation, carbon and energy metabolism, amino acid metabolism, oxidative phosphorylation, and lipid metabolism were the main metabolic pathways. Nitrogen, phosphorus, and carbon metabolism were closely related to the growth of *V. volvacea*, which is of great significance for understanding the growth and development of *V. volvacea* during the cultivation process.

## Data Availability

The original contributions presented in this study are included in the article. Further inquiries can be directed to the corresponding author.

## References

[1] Raman J., Jang K.Y., Oh Y.L., Oh M., Im J.H., Lakshmanan H., Sabaratnam V. (2021). Cultivation and Nutritional Value of Prominent Pleurotus Spp.: An Overview. Mycobiology.

[2] Pardo-Giménez A., Carrasco J., Roncero J.M., Alvarez-Ortí M., Zied D.C., Pardo-González J.E. (2018). Recycling of the biomass waste defatted almond meal as a novel nutritional supplementation for cultivated edible mushrooms. Acta Sci. Agron..

[3] Bao D.P., Gong M., Zheng H.J., Chen M.J., Zhang L., Wang H., Jiang J.P., Wu L., Zhu Y.Q., Zhu G. (2013). Sequencing and Comparative Analysis of the Straw Mushroom (*Volvariella volvacea*) Genome. PLoS ONE.

[4] Queiroz E.C., Marino R.H., da Eira A.F. (2004). Mineral supplementation and productivity of the shitake mushroom on Eucalyptus logs. Sci. Agric..

[5] Krishnappa B.G., Gowda P.A., Shivakumar S.P., Mallesha B.C., Raghunandan B.L., Divya M. (2014). Growth and Yield of Hypsozygous ulmarius Mushroom on Different Substrates Mixtures. J. Pure Appl. Microbiol..

[6] Zhu H., Zhao S.J., Jin A.A., Tang J.Y., Luo Y.Q. (2021). The use of un-composted spent mushroom residue as a replacement of peat in substrates for *Gossypium herbaceum* and *Talinum paniculatum*. Not. Bot. Horti Agrobot. Cluj-Napoca.

[7] Tie J.Z., Qiao Y.L., Jin N., Gao X.Q., Liu Y.Y., Lyu J., Zhang G.B., Hu L.L., Yu J.H. (2023). Yield and Rhizosphere Soil Environment of Greenhouse Zucchini in Response to Different Planting and Breeding Waste Composts. Microorganisms.

[8] Roger-Estrade J., Anger C., Bertrand M., Richard G. (2010). Tillage and soil ecology: Partners for sustainable agriculture. Soil Tillage Res..

[9] Tao R., Liang Y.C., Wakelin S.A., Chu G.X. (2015). Supplementing chemical fertilizer with an organic component increases soil biological function and quality. Appl. Soil Ecol..

[10] Rahmann S., Martin M., Schulte J.H., Köster J., Marschall T., Schramm A. (2013). videntifying transcriptional miRNA biomarkers by integrating high-throughput sequencing and real-time PCR data. Methods.

[11] Chen S.F., Zhou Y.Q., Chen Y.R., Gu J. (2018). fastp: An ultra-fast all-in-one fastq preprocessor. BioinFormatics.

[12] Agustinho D.P., Fu Y., Menon V.K., Metcalf G.A., Treangen T.J., Sedlazeck F.J. (2024). Unveiling microbial diversity: Harnessing long-read sequencing technology. Nat. Methods.

[13] Li D.H., Liu C.M., Luo R.B., Sadakane K., Lam T.W. (2015). Megahit: An ultra-fast single-node solution for large and complex metagenomics assembly via succinct *de Bruijn* graph. Bioinformatics.

[14] Steinegger M., Söding J. (2017). MMseqs2 enables sensitive protein sequence searching for the analysis of massive data sets. Nat. Biotechnol..

[15] Zhu W.H., Lomsadze A., Borodovsky M. (2010). *Ab initio* gene identification in metagenomic sequences. Nucleic Acids Res..

[16] Artsa P., van der Raadt J., van Gestel S.H.C., Steehouwer M., Shendure J., Hoischen A., Albers C.A. (2017). Quantification of differential gene expression by multiplexed targeted resequencing of cDNA. Nat. Commun..

[17] Bu D.C., Luo H.T., Huo P.P., Wang Z.H., Zhang S., He Z.H., Wu Y., Zhao L.H., Liu J.J., Guo J.C. (2021). KOBAS-i: Intelligent prioritization and exploratory visualization of biological functions for gene enrichment analysis. Nucleic Acids Res..

[18] Flinn K.M., Lechowicz M.J., Waterway M.J. (2008). Plant Species Diversity and composition of wetlands within An upland forest. Am. J. Bot..

[19] Buttigieg P.L., Ramette A. (2014). A guide to statistical analysis in microbial ecology: A community-focused, living review of multivariate data analyses. Fems Microbiol. Ecol..

[20] Wang H.C., Lin S.Y., Zhang H., Guo D., Liu D., Zheng X.W. (2023). Batch-fed composting of food waste: Microbial diversity characterization and removal of antibiotic resistance genes. Bioresour. Technol..

[21] Jordan S.N., Farrell M.P., Stephens C.T.G. (2025). Microbial community response to spent mushroom substrate composting methods by phospholipid fatty acid analysis. Biocatal. Agric. Biotechnol..

[22] Wu S., Zhou R.R., Ma Y.T., Fang Y., Xie G.P., Gao X.Z., Xiao Y.Z., Liu J.J., Fang Z.M. (2021). Development of a consortium-based microbial agent beneficial to composting of distilled grain waste for Pleurotus ostreatus cultivation. Biotechnol. Biofuels.

[23] Zhang X., Zhong Y.H., Yang S.D., Zhang W.X., Xu M.Q., Ma A.Z., Zhuang G.Q., Chen G.J., Liu W.F. (2014). Diversity and dynamics of the microbial community on decomposing wheat straw during mushroom compost production. Bioresour. Technol..

[24] Vajna B., Nagy A., Sajben E., Manczinger L., Szijártó N., Kádár Z., Bordás D., Márialigeti K. (2009). Microbial community structure changes during oyster mushroom substrate preparation. Appl. Microbiol. Biotechnol..

[25] Zhao G.Z., Liu C., Hadiatullah H., Yao Y.P., Lu F.P. (2021). Effect of *Hericium erinaceus* on bacterial diversity and volatile flavor changes of soy sauce. LWT.

[26] Phithakrotchanakoon C., Mayteeworakoon S., Siriarchawatana P., Kitikhun S., Harnpicharnchai P., Wansom S., Eurwilaichitr L., Ingsriswang S. (2022). Beneficial bacterial-Auricularia cornea interactions fostering growth enhancement identified from microbiota present in spent mushroom substrate. Front. Microbiol..

[27] Math R.K., Islam S.M.A., Hong S.J., Cho K.M., Kim J.M., Yun M.G., Cho J.J., Kim E.J., Lee Y.H., Yun H.D. (2010). Metagenomic Characterization of Oyster Shell Dump Reveals Predominance of *Firmicutes* Bacteria. Microbiology.

[28] Wang P., Ma J., Wang Z., Jin D.C., Pan Y.T., Su Y.Z., Sun Y., Cernava T., Wang Q. (2022). Di-n-butyl phthalate negatively affects humic acid conversion and microbial enzymatic dynamics during composting. J. Hazard. Mater..

[29] Lin S.S., Wang Y., Lin J.F., Wang X.R., Gong H.L. (2009). Dominant bacteria correlated with elimination of sludge in an innovative reactor. Prog. Nat. Sci..

[30] Belewu M.A., Belewu K.Y. (2005). Cultivation of mushroom (*Volvariella volvacea*) on banana leaves. Afr. J. Biotechnol..

[31] Zhang B., Yan L.J., Li Q., Zou J., Tan H., Tan W., Peng W.H., Li X.L., Zhang X.P. (2018). Dynamic succession of substrate-associated bacterial composition and function during Ganoderma lucidum growth. PeerJ.

[32] Hu X.J., Cao Y.C., Zhao X., Su H.C., Wen G.L., Yang Y.F. (2023). Effect of bacterial community succession on environmental factors during litter decomposition of the seaweed *Gracilaria lemaneiformis*. Mar. Pollut. Bull..

[33] Wang Z.C., Hou Q., Ahmed H.G.M.D., Akram M.I., Iqbal R., Al-Ghamdi A.A., Al Farraj D.A., Yang T., Kong C. (2025). Effect of different fertilization combinations on chinese cabbage quality, Amino acid content, and rhizosphere microorganisms. Appl. Ecol. Environ. Res..

[34] Balakrishnan K., Krishnaa D., Balakrishnan G., Manickam M., Abdulkader A.M., Dharumadurai D. (2024). Association of Bacterial Communities with Psychedelic Mushroom and Soil as Revealed in 16S rRNA Gene Sequencing. Appl. Biochem. Biotechnol..

[35] Liu N.Y., Liu Z.Z., Wang K.Y., Zhao J.F., Fang J., Liu G., Yao H., Pan J.T. (2024). Comparison analysis of microbial agent and different compost material on microbial community and nitrogen transformation genes dynamic changes during pig manure compost. Bioresour. Technol..

[36] Suwannarach N., Kumla J., Zhao Y., Kakumyan P. (2022). Impact of Cultivation Substrate and Microbial Community on Improving Mushroom Productivity: A Review. Biology.

[37] Carrasco J., Zied D.C., Pardo J.E., Preston G.M., Pardo-Giménez A. (2018). Supplementation in mushroom crops and its impact on yield and quality. AMB Express.

[38] Yang F.C., Hsieh C.Y., Chen H.M. (2003). Use of stillage grain from a rice-spirit distillery in the solid state fermentation of Ganoderma lucidum. Process Biochem..

[39] Peksen A., Yakupoglu G. (2009). Tea waste as a supplement for the cultivation of Ganoderma lucidum. World J. Microbiol. Biotechnol..

[40] Ozcelik E., Peksen A. (2007). Hazelnut husk as a substrate for the cultivation of shiitake mushroom (*Lentinula edodes*). Bioresour. Technol..

[41] Tao Z.D., Liu X.C., Sun L.L., He X.X., Wu Z.S. (2022). Effects of two types nitrogen sources on humification processes and phosphorus dynamics during the aerobic composting of spent mushroom substrate. J. Environ. Manag..

[42] Liu L.H., Cui S.Z., Qin M., Chen L.Q., Yin D.W., Guo X.H., Li H.Y., Zheng G.P. (2022). Effects of Continuous Ridge Tillage at Two Fertilizer Depths on Microbial Community Structure and Rice Yield. Agriculture.

[43] Li F.L., Kong Q.B., Zhang Q., Wang H.P., Wang L.M., Luo T. (2020). Spent mushroom substrates affect soil humus composition, microbial biomass and functional diversity in paddy fields. Appl. Soil Ecol..

[44] Wang H.W., Xu M., Cai X.Y., Tian F. (2021). Evaluation of soil microbial communities and enzyme activities in cucumber continuous cropping soil treated with spent mushroom (*Flammulina velutipes*) substrate. J. Soils Sediments.

[45] Alborés S., Pianzzola M.J., Soubes M., Cerdeiras M.P. (2006). Biodegradation of agroindustrial wastes by Pleurotus spp for its use as ruminant feed. Electron. J. Biotechnol..

[46] Noshad M., Behbahani B.A., Jooyandeh H., Rahmati-Joneidabad M., Kaykha M.E.H., Sheikhjan M.G. (2021). Utilization of Plantago major seed mucilage containing Citrus limon essential oil as an edible coating to improve shelf-life of buffalo meat under refrigeration conditions. Food Sci. Nutr..

[47] Kertesz M.A., Thai M. (2018). Compost bacteria and fungi that influence growth and development of *Agaricus bisporus* and other commercial mushrooms. Appl. Microbiol. Biotechnol..

[48] Hao H.B., Yue Y.H., Chen Q., Yang Y., Kuai B.K., Wang Q., Xiao T.T., Chen H., Zhang J.J. (2024). Effects of an efficient straw decomposition system mediated by Stropharia rugosoannulata on soil properties and microbial communities in forestland. Sci. Total Environ..

[49] Li X.R., Luo L., Wang X.Y., Zhu M. (2025). Further insights into the molecular mechanisms underlying tobacco straw cultivation of Pleurotus ostreatus by comparative transcriptome analyses. Genomics.

[50] Liu D., He X.H., Chater C.C.C., Perez-Moreno J., Yu F.Q. (2021). Microbiome Community Structure and Functional Gene Partitioning in Different Micro-Niches Within a Sporocarp-Forming Fungus. Front. Microbiol..

[51] Guo Y.X., Chen Q.J., Qin Y., Yang Y.R., Yang Q.Z., Wang Y.X., Cheng Z.A., Cao N., Zhang G.Q. (2021). Succession of the microbial communities and function prediction during short-term peach sawdust-based composting. Bioresour. Technol..

[52] Li D., Wang D., Fang Y.D., Belwal T., Li L., Lin X.Y., Xu Y.Q., Chen H.J., Zhu M., Luo Z.S. (2021). Involvement of energy metabolism and amino acid metabolism in quality attributes of postharvest *Pleurotus eryngii* treated with a novel phase change material. Postharvest Biol. Technol..

[53] Xiao C., Wu Q.P., Xie Y.Z., Tan J.B., Ding Y.R., Bai L.J. (2018). Hypoglycemic mechanisms of *Ganoderma lucidum* polysaccharides F31 in db/db mice via RNA-seq and iTRAQ. Food Funct..

[54] Wang L., Dong Q., Guo Q., Zha L., Yang L., Yu C.X., Zhao Y. (2025). Dynamics of Nutrient Components and Microbial Communities in Substrates During the Development of the Fruiting Bodies of *Volvariella volvacea*. J. Fungi.

[55] Zhao K., Jia X.B., Lin J.J., Zhao J., Lin C.Q., Chen J.C. (2024). Comparing the Promoting Effect of Constructed Bacterial Agents and Mature Compost on Chicken Manure Composting. Waste Biomass Valorization.

